# Isolation and characterisation of a ruminant alphaherpesvirus closely related to bovine herpesvirus 1 in a free-ranging red deer

**DOI:** 10.1186/1746-6148-3-26

**Published:** 2007-09-28

**Authors:** Julien Thiry, Frederik Widén, Fabien Grégoire, Annick Linden, Sándor Belák, Etienne Thiry

**Affiliations:** 1 Virology and Viral Diseases, Department of Infectious and Parasitic Diseases, Faculty of Veterinary Medicine, University of Liège, B-4000 Liège, Belgium; 2 Joint R&D Division, Departments of Virology, National Veterinary Institute and Swedish University of Agricultural Sciences, SE-75189 Uppsala, Sweden; 3 Bacteriology, Department of Infectious and Parasitic Diseases, Faculty of Veterinary Medicine, University of Liège, B-4000 Liège, Belgium

## Abstract

**Background:**

The genus *Varicellovirus *of the *Herpesviridae *subfamily *Alphaherpesvirinae *includes a cluster of viruses antigenically and genetically related to bovine herpesvirus 1 (BoHV-1): namely bovine herpesvirus 5 (BoHV-5), bubaline herpesvirus 1 (BuHV-1), caprine herpesvirus 1 (CpHV-1), cervid herpesviruses 1 (CvHV-1) and 2 (CvHV-2) and elk herpesvirus 1 (ElkHV-1). Considering the serological relationship between these ruminant alphaherpesviruses, several surveys have studied the occurrence of BoHV-1 related virus infection in wild and domestic ruminant species. In this way, a recent investigation has indicated, in Belgium, a high increase in the serological prevalence of BoHV-1 related virus infection in free-ranging red deer population. In this context, it has been decided to investigate the presence of an alphaherpesvirus spreading in the Belgian free-ranging red deer population.

**Results:**

The current study reports the first isolation in a free-ranging red deer of a BoHV-1 closely related virus. The isolate was antigenically, genomically and genetically characterised by comparison with several ruminant alphaherpesvirus. Immunofluorescence assays revealed the isolate was antigenically distinct from bovine and caprine alphaherpesviruses. Similarly, BamHI and BstEII restriction analyses demonstrated the genomic difference between the isolate and the other ruminant alphaherpesviruses. Next, the sequencing of selected parts of UL27 and US8 genes showed a high degree of homologies between each BoHV-1 related ruminant alphaherpesvirus and the isolate. Besides the close relationship between all ruminant alphaherpesviruses, the phylogenetic analysis revealed that the isolate clustered with CvHV-1.

**Conclusion:**

The first isolation of a virus closely related to BoHV-1 in a free-ranging red deer is reported. Data demonstrate that a CvHV-1 strain, named Anlier, circulates in wild red deer in continental Europe. Anlier strain show consistent differences with the virus isolated from Scottish farmed red deer. All together, these results improve our understanding of ruminant alphaherpesviruses.

## Background

The family *Herpesviridae *includes nearly two hundred viruses isolated from various hosts including molluscs, fishes, amphibians, reptiles, birds, mammals and at least one invertebrate. Based on biological and molecular properties, the family has been divided into three subfamilies of viruses, *Alpha-*, *Beta- *and *Gammaherpesvirinae*, which have co-evolved with different host species. Illustrating the concept, several ruminant alphaherpesviruses form a cluster of antigenically and genetically related viruses [1]. Seven alphaherpesviruses belong to this cluster where bovine herpesvirus 1 (BoHV-1), responsible for infectious bovine rhinotracheitis (IBR), a cattle disease of major economic concern in Europe, is the prototype [2] : bovine herpesvirus 5 (BoHV-5) causing meningo-encephalitis in calves [3], bubaline herpesvirus 1 (BuHV-1) responsible for subclinical infections in water buffaloes [4], caprine herpesvirus 1 (CpHV-1) inducing systemic disease in kids and abortion in adults [5], cervid herpesvirus 1 (CvHV-1) responsible for conjunctivitis in red deer [6], cervid herpesvirus 2 (CvHV-2) and elk herpesvirus 1 (ElkHV-1) causing subclinical genital infections in reindeer and elk respectively [7,8]. Phylogenetic studies of conserved herpesvirus sequences showed that BoHV-5 and BuHV-1 were most closely related to BoHV-1, followed by ElkHV-1, CvHV-1, CvHV-2 and CpHV-1 [7,9,10]. However, BoHV-1 related ruminant alphaherpesvirus are not always restricted to their natural host species. Indeed, buffalo, goat, sheep, red deer and reindeer were successfully infected with BoHV-1 under experimental conditions. Similarly, cattle were shown to be susceptible to BuHV-1, CpHV-1, CvHV-1, CvHV-2 and ElkHV-1 [1]. The cross-serological relationship between these viruses and BoHV-1 was also demonstrated by seroneutralisation and enzyme linked immunosorbent assays (ELISA) [11-14]. Consequently, the properties shared by BoHV-1 related alphaherpesviruses can lead to misdiagnosis of BoHV-1 infection which can be considered as a threat to infectious bovine rhinotracheitis eradication programmes [1].

CvHV-1 was firstly isolated in 1983 from a farmed red deer suffering of ocular lesions. The disease, showing a contagious character, emerged at the end of 1982 in a red deer stag in northern Scotland. Fifty to sixty animals out of 80 exhibited clinical signs at various degrees. A seroneutralisation assay demonstrated the serological relationship of the virus with BoHV-1 [6]. Since this isolation, no epidemic of the ocular disease in red deer stags and no severe epidemic in free-ranging animals have been reported. More recently, CvHV-1 was identified in New-Zealand during routine export examination of semen collected from red deer stags [15]. CvHV-1 is responsible for the herpetic conjunctivitis of red deer commonly named ocular syndrome. The disease is characterised by purulent ocular discharge, hypopyon, uniform corneal opacity without ulceration, mucopurulent nasal discharge and photophobia. Moderate swelling of the periorbital tissues and marked oedema of the upper eyelids are also observed [6]. The reactivation of CvHV-1 was successfully performed suggesting the persistence of the infection in a latent state [16].

Since it was demonstrated that red deer is infected by a herpesvirus, several studies have been initiated to evaluate the percentage of animals seropositive to BoHV-1 and CvHV-1 [17,18]. The first serological survey revealed that CvHV-1 infection was widespread in Scotland with prevalences of 40% in hill deer and 33% in farmed deer [19]. English farmed deer were also tested for CvHV-1 antibody and presented a prevalence of 14% [19]. In Germany, 12.8% of red deer hunted between 1984 and 1986 were seropositive to BoHV-1 [20] and the serological evidence of a CvHV-1 infection was reported one year later [21]. More recently, antibodies against BoHV-1 were found in 5.4% red deer from the German region Brandenburg [22]. In Czech republic, 68% and 71% of red deer were seropositive to BoHV-1 and CvHV-1 respectively [23] and 0.5% of free-ranging red deer in Norway [24]. Outside of Europe, CvHV-1 infection was identified in New Zealand [15,25] and 55% of red deer living in United States National Parks were seropositive to BoHV-1 [26]. However, these results only suggest a red deer CvHV-1 infection since the selected tests were not specific to CvHV-1 but to BoHV-1 [1,27].

In Belgium, two serological investigations aiming to detect antibodies against BoHV-1 were performed. The first one indicated that 11% of Belgian red deer were seropositive to BoHV-1 or another related herpesvirus [28]. In 2001 and 2002, a higher prevalence was noticed with 28.9% of red deer positive to BoHV-1 (F. Gregoire and A. Linden, unpublished data). However, since experimental quantification of BoHV-1 transmission in red deer demonstrated that BoHV-1 would probably not survive more than a few decades in red deer populations [27], it is most likely that BoHV-1 antibodies prevalence was due to CvHV-1 and not a BoHV-1 cross-species infection. Regarding these data, it was decided to investigate the presence of an alphaherpesvirus spreading in the Belgian free-ranging red deer population.

## Results

### Isolation and identification of a red deer alphaherpesvirus

During the 2004 and 2005 hunting seasons, nasal and genital swabs were collected from free-living red deer in the South of Belgium. A viral isolate (050136) was obtained in MDBK cells inoculated with a nasal swab from a male fawn (*Cervus elaphus*) hunted in Anlier forest. The animal was in good shape and weighed 47 kg. The confirmation of the positive sample was performed by inoculation with the duplicate swab. The virus induced a cytopathic effect morphologically and temporally typical of a herpesvirus (data not shown). By PCR [10], a 443 bp region of glycoprotein B gene (UL27) was amplified (data not shown). Taken together, results suggested that the Anlier isolate belonged to the cluster of BoHV-1 related alphaherpesviruses. The analysis of the PCR product sequence showed that the Anlier isolate was close to BoHV-1 related ruminant alphaherpesviruses especially CvHV-1 (data not shown).

### Antigenic characterisation of the Anlier isolate within ruminant alphaherpesviruses

**Figure 1:**
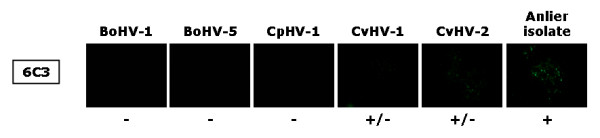
**Indirect immunofluorescence staining of MDBK cells infected with either BoHV-1, BoHV-5, CpHV-1, CvHV-1, CvHV-2, or the Anlier isolate**. Cells were incubated until viral plaques appeared and were then treated as described in Materials and Methods. 6C3 is the primary antibody and was detected by FITC-conjugated rabbit immunoglobulin anti-mouse IgG. Presence of viral plaques was checked by transmission microscopy before epifluorescence microscopy. All immunofluorescence stainings were performed three times. Symbols: +, positive signal; -, negative signal; +/-, weak signal.

In order to discriminate antigenically the Anlier isolate between bovine, caprine and cervid alphaherpesviruses, five selected monoclonal antibodies were tested by immunofluorescence assay on cells infected separately with the five related alphaherpesviruses: G14G11F5 for BoHV-1, 2915 for BoHV-5, 2E5G5G1 for CpHV-1, 6C2 for CvHV-1 and 5G10 for CvHV-2. Monoclonal antibodies detected their specific viruses as demonstrated by Keuser and collaborators [29], however, none of them reacted with the Anlier isolate. One can speculate that since CvHV-1 was also isolated from a red deer, CvHV-1 and the Anlier isolate might cross-react serologically. Therefore, other monoclonal antibodies were assessed to detect specifically the Anlier isolate. One monoclonal antibody, 6C3, identified the Anlier isolate. However, a weak reaction was also observed on CvHV-1 or CvHV-2 infected cells (Figure 1). Taken together, these results demonstrated that the Anlier isolate was antigenically distinct from BoHV-1, BoHV-5 and CpHV-1, but presumably possesses some common epitopes with CvHV-1 and CvHV-2.

### Genomic comparison of the Anlier isolate to ruminant alphaherpesviruses

**Figure 2:**
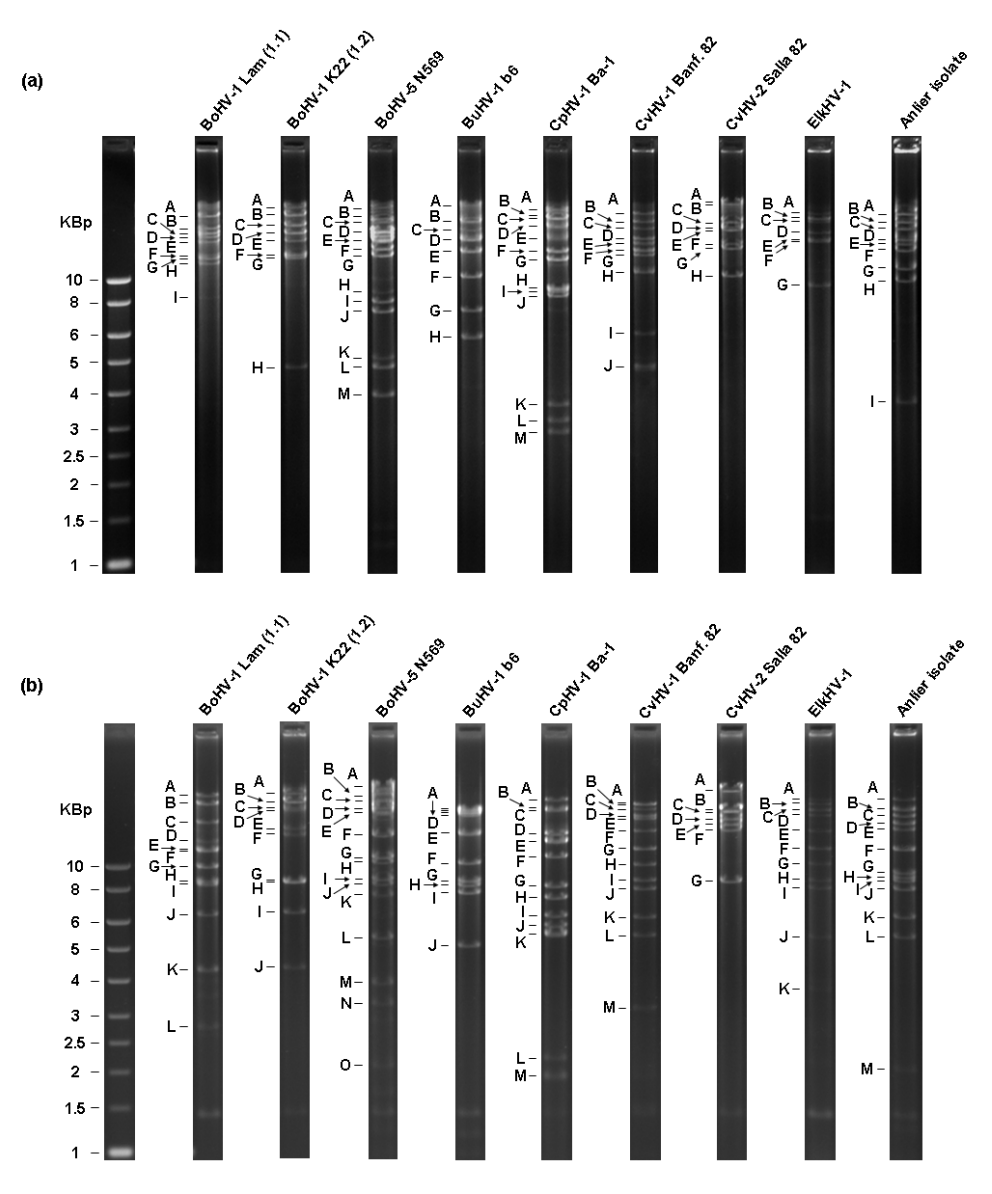
BamHI (a) and BstEII (b) restriction endonuclease profiles of the Anlier isolate and ruminant alphaherpesviruses.

To avoid any misinterpretation due to antigenic cross reactions, Anlier isolate and ruminant alphaherpesviruses were submitted to restriction enzyme analysis by using two endonucleases, BamHI (Figure 2a) and BstEII (Figure 2b). The patterns were in accordance with the previously published patterns [30-32] and clearly allowed a differentiation between each ruminant alphaherpesvirus as well as between BoHV-1 subtypes 1 and 2 (Figure 2). These data confirmed that the Anlier isolate was genomically distinct from the other related ruminant alphaherpesviruses.

### Phylogenetic relationship between Anlier isolate and ruminant alphaherpesviruses

**Figure 3:**
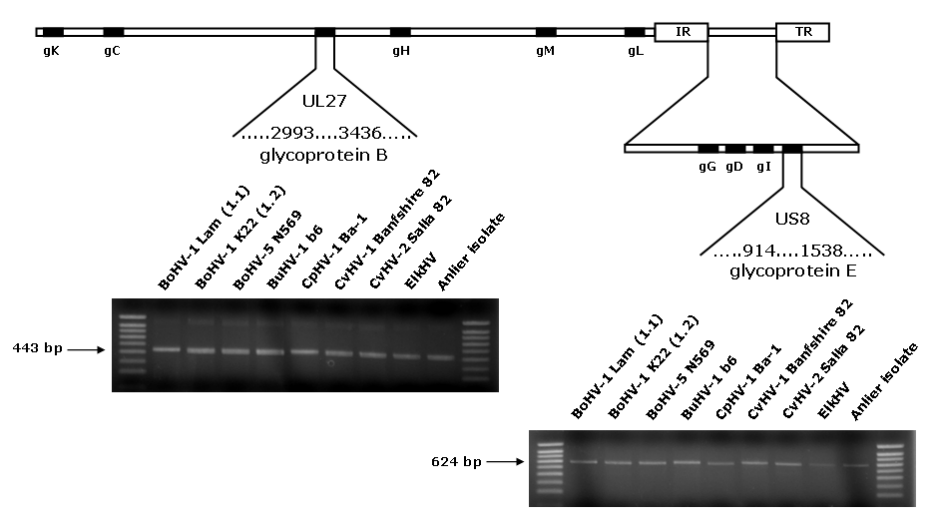
PCR amplifications of selected parts of UL27 and US8 sequences of the Anlier isolate and ruminant alphaherpesviruses.

**Figure 4:**
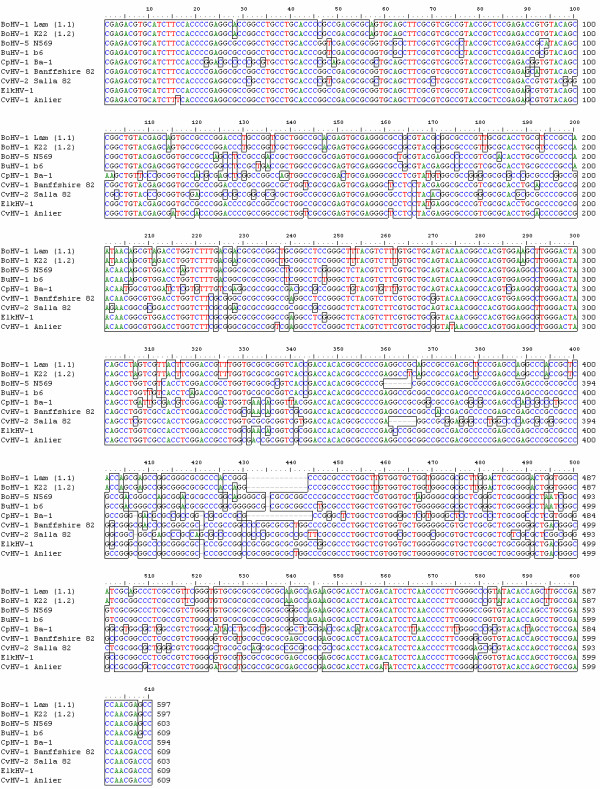
Alignment of a selected part of US8 sequence of the Anlier isolate and ruminant alphaherpesviruses.

**Figure 5:**
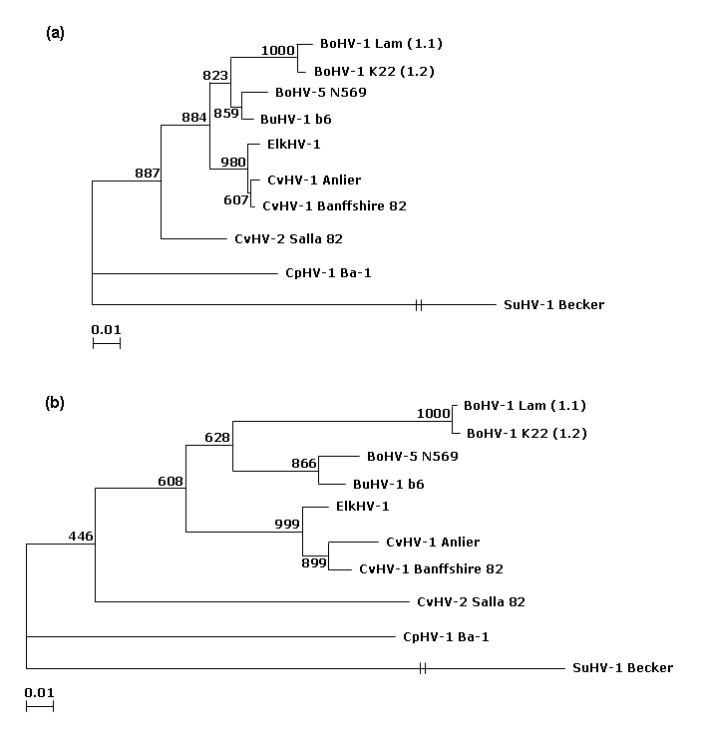
**Phylogenetic relationships between the Anlier isolate and ruminant alphaherpesviruses**. Trees were generated by using selected parts of UL27 (a) or US8 (b) nucleotide sequences, and were both rooted by using Suid herpesvirus 1 (SuHV-1) Becker strain gB sequence as an outgroup. The reliability of the trees were assesed by resampling and analysing 1000 random data sets (bootstrapping) showed at branches.

To further characterise the Anlier isolate, different regions of the genome were sequenced (Figure 3). Partial sequence data from UL27 encoding the glycoprotein B (gB) and US8 encoding the glycoprotein E (gE) were obtained for eight ruminant alphaherpesviruses including the two BoHV-1 subtypes and the Anlier isolate. These sequences represented two different degrees of conservation among ruminant alphaherpesviruses genes. The sequence lengths were 444 nucleotides for UL27 and varied between 597 and 609 nucleotides for US8. By analysing multiple nucleotide sequences alignments in the US8 sequence, CvHV-1 and CvHV-2 differed from each other by 96 out of 609 bases, CvHV-1 and ElkHV-1 by 15 out of 609 bases, ElkHV-1 and the Anlier isolate by 22 out of 609 bases, CvHV-2 and the Anlier isolate by 99 out of 609 bases, and CvHV-1 and the Anlier isolate by 16 out of 609 bases (Figure 4). Tables 1 and 2 compare the percentage of nucleotide and amino acid sequence identity between each ruminant alphaherpesviruses. Phylogenetic analyses were also performed to assess the relationship between the Anlier isolate and other ruminant alphaherpesviruses (Figure 5). BoHV-5 and BuHV-1 clustered together and are the most closely related to BoHV-1 1.1 and BoHV-1 1.2. CpHV-1 is the most diverging ruminant alphaherpesvirus. CvHV-1 is more related to BoHV-1 than ElkHV-1 and CvHV-2 which is the most closely related to CpHV-1. The Anlier isolate clustered with CvHV-1 and the most closely related virus is ElkHV-1. The same topography was obtained for phylogenetic trees based on UL27 (Figure 5a) and US8 nucleotide sequences (Figure 5b). Taken together, these data revealed high degrees of homology between each ruminant alphaherpesvirus, the highest being observed between CvHV-1 and the Anlier isolate demonstrating the identification of a new CvHV-1 strain.

**Table 1:** UL27 nucleotide and amino acid sequence similarities between the Anlier isolate and ruminant alphaherpesviruses

	**Nucleotide similarity**										
	**UL27**	**BoHV-1 Lam (1.1)**	**BoHV-1 K22 (1.2)**	**BoHV-5 N569**	**BuHV-1 b6**	**CpHV-1 Ba-1**	**CvHV-1 Banf. 82**	**CvHV-2 Salla 82**	**ElkHV-1**	**CvHV-1 Anlier**	**SuHV-1 Becker**
**Amino acid similarity**	**BoHV-1 Lam (1.1)**		99	95.9	96.6	87.8	94.8	92.3	94.5	94.8	75.4
	**BoHV-1 K22 (1.2)**	100		95.7	96.1	87.8	94.5	92.1	94.5	94.5	75.2
	**BoHV-5 N569**	97.9	97.9		98.6	88.5	96.3	93.6	95.9	96.1	76.3
	**BuHV-1 b6**	97.2	97.2	99.3		88.9	96.8	93.6	96.6	96.8	76.5
	**CpHV-1 Ba-1**	88.5	88.5	88.5	87.8		88	88.7	88.2	87.8	75.6
	**CvHV-1 Banf. 82**	94.5	94.5	95.2	95.9	87.8		95	99.3	99.3	76.5
	**CvHV-2 Salla 82**	91.8	91.8	91.8	91.8	88.5	94.5		95.2	94.3	77.7
	**ElkHV-1**	94.5	94.5	94.5	94.5	88.5	98.6	95.9		98.8	75.9
	**CvHV-1 Anlier**	94.5	94.5	95.2	95.2	86.4	98.6	93.2	97.2		75.9
	**SuHV-1 Becker**	70.9	70.9	70.2	70.2	71.6	69.5	70.2	68.9	68.2	

Suid herpesvirus 1 (SuHV-1) Becker strain was used as an outgroup.

**Table 2:** US8 nucleotide and amino acid sequence similarities between the Anlier isolate and ruminant alphaherpesviruses

	**Nucleotide similarity**										
	**US8**	**BoHV-1 Lam (1.1)**	**BoHV-1 K22 (1.2)**	**BoHV-5 N569**	**BuHV-1 b6**	**CpHV-1 Ba-1**	**CvHV-1 Banf. 82**	**CvHV-2 Salla 82**	**ElkHV-1**	**CvHV-1 Anlier**	**SuHV-1 Becker**
**Amino acid similarity**	**BoHV-1 Lam (1.1)**		99.4	85.3	86.5	76.2	83.4	76.5	83.7	83.1	50.2
	**BoHV-1 K22 (1.2)**	99		85	86.6	76.3	83.6	76.5	83.9	83.2	50
	**BoHV-5 N569**	76.4	75.9		96.5	74.8	87.3	80.6	88.3	87	53.9
	**BuHV-1 b6**	78.8	79.8	92.7		76.8	89	80.9	90	88.8	54
	**CpHV-1 Ba-1**	59.7	60.1	59.3	61.2		77.8	76.3	78.6	77.8	50.2
	**CvHV-1 Banf. 82**	69.8	70.8	76.4	79.8	60.2		82.6	97.5	97.3	53.2
	**CvHV-2 Salla 82**	64.5	65	67.3	69.8	60.2	70.3		82.7	82.4	52.9
	**ElkHV-1**	69.8	70.8	77.8	81.2	61.2	94.2	70.8		96.2	53.9
	**CvHV-1 Anlier**	70.3	71.2	76.4	80.2	57.8	95.1	69.8	92.3		52.4
	**SuHV-1 Becker**	25	25	30.4	30.7	25.8	28.7	25.3	29.1	27.2	

Suid herpesvirus 1 (SuHV-1) Becker strain was used as an outgroup.

## Discussion

The use of a consensus PCR detecting bovine, caprine, red deer and reindeer alphaherpesviruses [10] has allowed the detection and the identification of the CvHV-1 Anlier strain and its phylogenic position in the BoHV-1 related alphaherpesvirus cluster. No cross-reaction was observed when monoclonal antibodies specific of ruminant alphaherpesviruses were applied to cells infected with the Anlier strain. In contrast, the monoclonal antibody 6C3, described as being specific to CvHV-1 [29], surprisingly detected more specifically the Anlier strain than CvHV-1. The weak reactivity observed with CvHV-2 could result from epitopes common with the Anlier strain. To further characterise the Anlier strain in comparison with ruminant alphaherpesviruses, restriction enzyme analysis were performed. Similarities in the BamHI and BstEII restriction patterns further support that the Anlier isolate is a new strain of CvHV-1.

When analysing nucleotide and amino acid sequences similarities, the difference in the degree of conservation between UL27 and US8 sequences is demonstrated among BoHV-1 related ruminant alphaherpesviruses, confirming BoHV-5 previously obtained data [33]. Such difference in the level of conservation between UL27 and US8 sequences could influence the diagnosis of ruminant alphaherpesvirus infections by both BoHV-1 gB and gE blocking ELISAs. Indeed, experimentally BoHV-5 infected calves were shown to be positive in BoHV-1 gB blocking ELISA and negative in BoHV-1 gE blocking ELISA allowing the discrimination between BoHV-1 and BoHV-5 [11]. As the highest US8 sequences similarity is observed between BoHV-1 and BoHV-5, it can be hypothesised such methodology serologically discriminate between other heterologous infections, e.g. between BoHV-1 and CpHV-1 infection of goats [34] or between BoHV-1 and CvHV-1 infection in red deer [27].

Phylogenetic analysis showed that the most closely related viruses to BoHV-1 1.1 and BoHV-1 1.2 are BoHV-5 and BuHV-1 followed by CvHV-1 and ElkHV-1 clustering together and further by CvHV-2 and CpHV-1. These results extend the knowledge on the genetic relatedness of BoHV-1 related ruminant alphaherpesviruses to BuHV-1 and ElkHV-1 [9,10,35-37]. Each ruminant alphaherpesvirus is closely related to each other both for UL27 and US8 sequences similarities confirming the presence of a consistent group of ruminant alphaherpesviruses in the family *Herpesviridae*. Additionally, primers designed to partially amplify US8 bring a new tool in the detection of ruminant alphaherpesvirus infection. Furthermore, they could be used to differentiate wild type BoHV-1 from vaccinal gE-negative BoHV-1 [38].

Genetic analysis of the Anlier strain reveals clear differences with CvHV-1 reference strain (16 out of 609 bases for US8 and 3 out of 444 bases for UL27). However, nothing is known about the potential strain pathogenicity. Moreover, deeper investigations are necessary to assess if this red deer alphaherpesvirus is a new viral subtype. Experimental infection of red deer with CvHV-1 lead to mild clinical signs, such as hyperthermia, nasal ulceration and conjunctivitis, and virus was reisolated from nasal and ocular swabs [16,39]. Natural infection of farmed red deer induced an ocular syndrome associated with various clinical manifestations [6]. Here, the Anlier strain was isolated from a nasal swab and the animal did not present any lesions. Moreover, no case of suspicious red deer death was noticed during 2004 and 2005 hunting seasons although a high seroprevalence was observed (F. Gregoire and A. Linden, unpublished data). Based on these data, it can be postulated that Anlier strain as several other herpesviruses could induce subclinical respiratory infection. However, it can be also hypothesised that as other herpesviruses too, it may predispose the respiratory tract for secondary bacterial infections or has the capacity by itself to initiate other disease forms [2]. In order to answer these questions, Anlier strain pathogenicity should be investigated by red deer experimental inoculation.

Belgian CvHV-1 emergence might be explained by red deer translocation in Europe. The biogeographic history of European red deer is under human influence [40,41]. During the last one thousand years, there was an extensive trade of the species mainly with the aim to improve the hunted trophy quality [40,42]. The existence of illegal translocation of animals by hunters was recently evidenced in Luxemburg. By using a multilocus genotyping, a red deer group was excluded from the autochthonous population [43] suggesting the presence imported red deer from foreign countries. In the Walloon Region of Belgium, reintroduction of imported animals in wildlife is unclear due to a lack of documentation. An extinction of the species occurred in 1848 and red deer were most likely imported from eastern European countries. In this regard, genetic analysis showed that Scottish CvHV-1 Banffshire 82 strain is different from Belgian Anlier strain. Consequently, viral transmission to a Belgian red deer from a reintroduced Scottish farmed red deer can not be currently supported in absence of molecular epidemiology of the two strains.

In contrast, the hypothesis of an endemic infection occurring in continental Europe has to be considered. A genetic cluster of red deer is distributed from Belgium, across Luxemburg, to Germany [43]. Similarly, the presence of genetic units over larger area has also been proven for the Engadin Valley population in eastern Switzerland [44] and in the Carpathian mountains in Romania [45]. It can be hypothesised that animals infected with Anlier strain belong to red deer genetic clusters described in the Czech Republic, Germany, and Hungary and spread the infection. This hypothesis is supported by the high BoHV-1 seroprevalence observed in red deer in these countries [20-23]. In Scotland, the wild red deer herds have produced the initial stocks for many deer farms [46] where the Scottish CvHV-1 Banffshire 82 strain was isolated [6]. This strain was probably already established in the wild deer population in Great Britain. As most British populations are non-indigenous [40,41], the virus could have been introduced a long time ago in the red deer population in Great Britain from continental Europe and could have diverged from the original virus. The virus could have persisted in small populations because of latency [16] and the industrial deer farming facilitated the identification of the virus. However, additional experiments are requested to investigate if the Belgian Anlier strain could be the ancestor of the Scottish Banffshire 82 strain.

## Conclusion

According to our knowledge, this is the first report on the isolation of a ruminant alphaherpesvirus from free-ranging red deer. The antigenic, genomic and genetic analysis demonstrated that the detected virus, termed CvHV-1 Anlier strain, is closely related to, but different from the CvHV-1 Banffshire 82 strain. This isolation and our knowledge of red deer population evolution in Europe allow the formulation of an interesting hypothesis on the origin of this virus in red deer knowing CvHV-1 Banffshire 82 strain had been isolated in farmed red deer in Scotland. Furthermore, our investigation brings the first comparative diagnosis of all known ruminant alphaherpesviruses related to BoHV-1 and especially a genetic definition of these viruses.

## Methods

### Cells and viruses

The Madin-Darby bovine kidney (MDBK) cell line (ATCC CCL22) was maintained in Earle minimal essential medium (MEM) (Invitrogen, Merelbeke, Belgium) supplemented with 5% of heat-inactivated foetal bovine serum (FBS) (BioWhittaker, Verviers, Belgium) and 2% penicillin (5,000 Units/ml) streptomycin (5,000 μg/ml) (PS) (Invitrogen). The CvHV-1 virus Anlier strain and BoHV-1 Lam 1.1 [47], BoHV-1 K22 1.2a [48], BoHV-5 N569 [49], BuHV-1 b6 [4], CpHV-1 Ba-1 [50], CvHV-1 Banffshire 82 [6], CvHV-2 Salla 82 [51], ElkHV-1 [7] virus strains were propagated on MDBK cells in MEM supplemented with 5% FBS. Viral stocks were produced by infection of confluent MDBK cells at a multiplicity of infection (MOI) of 0.1 in MEM supplemented with 2% PS and 5% FBS. When the cytopathic effect reached 90%, culture medium was removed and clarified by centrifugation at 1,500 × *g *for 20 min. The supernatants were aliquoted, frozen at -80°C, and titrated by plaque assay on MDBK cells as previously described [52].

### Viral DNA extraction

Viruses propagated on MDBK cells were clarified by centrifugation at 1,500 × *g *for 20 min. Supernatants were removed and further ultracentrifuged at 100,000 × *g *for 60 min. to pellet virions. Pellets were resuspended in TE buffer (10 mM TrisHCl pH 7.8, 1 mM EDTA) with 0.1% NP40, incubated 30 min at 37°C and ultracentrifuged 2 hours at 100,000 × *g *through a 30% (w/v) sucrose cushion. Pellets were resuspended in TE buffer with 0.5% sodium dodecylsulfate (SDS) and digested with proteinase K (700 μg/ml, Roche Diagnostic) for 2 h. at 56°C. Proteins were precipitated by the addition of half volume ammonium acetate (1.5 M, pH 7.5) and DNA was precipitated by addition of 2 volumes of ethanol. Viral DNA was extracted as previously described [52].

### Restriction enzyme analysis

Viral DNA was submitted to BamHI and BstEII restriction endonucleases (New England Biolabs, England, United Kingdom). The digestion products were electrophoresed in a 0.7% Tris Acetate EDTA gel for 18 h at 40 V/cm and 500 mA. SmartLadder (10 kb; Eurogentec, Liège, Belgium) was used as molecular mass marker.

### Immunofluorescence staining

An immunofluorescence assay was performed as described by Keuser and collaborators [29] with minor modifications. Briefly, MDBK cells grown on glass coverslips were infected with the different viruses and incubated 48 h in MEM containing 5% FBS and 0.6% carboxymethylcellulose. The coverslips with individual plaques were fixed in PBS containing 2% (wt/vol) paraformaldehyde and incubated with undiluted hybridoma supernatant or 1,000-fold-diluted ascitic fluid in PBSF. As secondary antibodies, FITC-conjugated rabbit anti-mouse IgG (2 μg/ml; Dako) was used. Coverslips were mounted with a Prolong Antifade kit (Molecular Probes Europe BV, Leiden, The Netherlands). Pictures were captured with a charge-coupled device Leica DC 300F camera (with Leica IM 50 V1.20 software) installed on an epifluorescence microscope.

### Sequencing and phylogenetic analysis

Two different sets of primers were used in this study. The diagnostic PCR and the sequencing of a 443 bp region of glycoprotein B gene (UL27) was performed by applying the CR30 (5'-TCGAARGCCGAGTACCTGCG-3'; sense; 5' end, position 56,051) and CR31 (5'-CCAGTCCCAGGCRACCGTCAC-3'; antisense; 5' end, position 56,494) primer set [10]. To extend phylogenetic analyses between ruminant alphaherpesviruses and the viral isolate, another set of primers amplifying an estimated 624 bp region of glycoprotein E gene (US8) was designed: ALPHA/US8/914F (5'-CGARACSTGCATCTTYCACC-3'; sense; 5' end, position) and ALPHA/US8/1538R (5'-GGSTCGTTGSTYGGM-3'; antisense; 5' end, position). Fragments resulting from PCR were purified with QIAquick PCR Purification Kit (Quiagen, Venlo, The Netherlands) and cloned using the pGEM^®^-T Easy vector system (Promega, Leiden, The Nederlands). The sequencing was performed by the GIGA sequencing facility (GIGA, Liège, Belgium). Sequences were assembled and aligned by using the BioEdit Sequence Alignment Editor software [53]. Phylogenetic analyses were performed, by distance methods, using the programs dnadist, neighbor and drawgram included in the PHYLIP package [54]. The reliability of the trees were assesed by resampling and analysing 1000 random data sets (bootstrapping) using Seqboot and consense software in the PHYLIP package.

### Nucleotide sequence accession number

The sequences reported in this paper have been deposited in the GenBank database under the accession numbers EF624466 to EF624479. The following previously GenBank published sequences were used in this study: BoHV-1 K22 UL27 [AF078725.1], BoHV-5 N569 UL27 [AF078726.2], CvHV-1 Banffshire 82 UL27 [AF078729.2] and CvHV-2 Salla 82 UL27 [AF078727.2].

## Competing interests

The author(s) declares that there are no competing interests.

## Authors' contributions

JT and ET designed the experiments, analysed the data and drafted the manuscript together. JT performed the experiments. JT and FG collected red deer samples. FW participated to the sequence alignment and constructed phylogenetic trees. SB and AL helped to draft the manuscript. All authors read and approved the final manuscript.
